# Utilization of eye health services and diabetic retinopathy: a cross-sectional study among persons living with diabetes visiting a tertiary eye care facility in Ghana

**DOI:** 10.1186/s12913-021-06594-y

**Published:** 2021-06-19

**Authors:** Bridgid Akrofi, John Tetteh, Kwesi N. Amissah-Arthur, Eileen N.A. Buxton, Alfred Yawson

**Affiliations:** 1 Eye Clinic of National Diabetes Management and Research Centre, Korle-Bu Teaching Hospital, Accra, Ghana; 2 Department of Community Health, College of Health Sciences, University of Ghana Medical School, University of Ghana, Accra, Ghana; 3 Department of Surgery (Eye), College of Health Sciences, University of Ghana Medical School, University of Ghana, Accra, Ghana; 4 Eye clinic department, Korle-Bu Teaching Hospital, Accra, Ghana

**Keywords:** Diabetes, Eye Care Health Service Utilization, Diabetic Retinopathy, Non-Proliferative Diabetic Retinopathy

## Abstract

**Background:**

There have been a major advance made in screening, early diagnosis, and prompt treatment of Diabetic Retinopathy among Person living with diabetes (PLWD). However, screening services remain a challenge in Low-Middle-Income-Countries where access to eye care professionals is inadequate. This study assesses the utilization of Eye Health Service prevalence (UEHS) among PLWD and associated factors and further quantifies its association with Non-Proliferative Diabetic Retinopathy (NPDR).

**Methods:**

A cross-sectional study design with a random sample of 360 PLWD was conducted at Korle-Bu Teaching Hospital, a National Referral Centre in Ghana from May to July 2019. UEHS and DR were the study outcomes. We adopted Poisson and Probit regression analysis to assess factors associated with UEHS over the past year. We employed pairwise and phi correlation (fourfold correlational analysis) to assess the relationship between UEHS and DR (ordinal and binary respectively). Ordered Logistic and Poisson regression were applied to assess the association between the UEHS and DR. Stata 16.1 was used to perform the analyses and a p-value ≤ 0.05 was deemed significant.

**Results:**

The prevalence of UEHS over the past year and DR was 21.7 %(95 %CI = 17.7–26.2) and 65.0 %(95 %CI = 59.9–69.8 respectively. The prevalence of severe NPDR with Clinically Significant Macular Edema (CSME) was 23.9 %(19.8–28.6). Type of diabetes, increasing age, educational level, mode of payment for healthcare services, marital status, years since diagnosis, and current blood glucose significantly influenced UEHS. There was a negative relationship between DR and UEHS (Pairwise and φ correlation were − 20 and − 15 respectively; p < 0.001). Non-UEHS among PLWD doubles the likelihood of experiencing severe NPDR with CSME compared with UEHS among PLWD [aOR(95 %CI) = 2.05(1.03–4.08)]. Meanwhile, the prevalence of DR among patients per non-UEHS was insignificantly higher [12 %; aPR(95 %CI) = 0.89–1.41)] compared with patients who utilized eye care health service.

**Conclusions:**

Most of the PLWD did not utilize the eye health service even once in a year and that was highly influenced by type of diabetes and increasing age. Type 2 diabetes patients and middle age decreased the likelihood of UEHS. There was a negative relationship between DR and UEHS among PLWD and this doubled the likelihood of experiencing severe NPDR with CSME. Structured health education and screening interventions are key to improving UEHS.

## Background

Diabetes mellitus is major public health problem which generally may cause vascular damage to the eyes, hearts, kidneys, and nerves, resulting in numerous complications [1]. Globally, the total estimated number of people living with diabetes has been estimated to increase from 382 million in 2013 to 592 million by 2035 with the majority occurring in Low-and-Middle-Income Countries (LMICs) [2]. In Africa, where diabetes was once rare, there has been a rise in the condition. It is estimated that about 39,000 people suffered from Type 1 diabetes in 2013 in Africa [3]. In Ghana, statistics on the prevalence of diabetes mellitus is scanty. The crude prevalence of diabetes in Ghana in 2002 was 6.3 % [4]. However, in 2012, a study by Danquah and colleagues estimated Type 2 diabetes prevalence rate of 97 % in Ghana urban settings among 1460 participants [5].

The risk of developing diabetic retinopathy (DR) is very high if diabetes is not well controlled [6–8]. The global prevalence of DR has been estimated to be 27 % with 33.8 % occurring in Africa [9]. There have been major advances made in screening, early diagnosis, and prompt treatment of DR among Person living with diabetes (PLWD), which allows for preventing about 98 % of visual challenges [10]. However, screening services remain a challenge in LMICs where access to eye care professionals and eye care services are inadequate [11].

In Ghana, accessibility to eye care professionals by patients with diabetes remains a challenge because the eye care Centres are not affiliated to the endocrine clinics, and this is evident at the three medical tertiary institutions in the country, namely; Korle Bu Teaching Hospital (KBTH), Komfo Anokye Teaching Hospital (KATH) and Tamale Teaching Hospital (TTH). Patients who utilize the eye care services are less likely to develop complications from DR than patients who do not utilize the eye clinic [12]. This study assesses Utilization of Eye Health Service prevalence (UEHS) among PLWD and associated factors and further quantify its association with Non-Proliferative Diabetic Retinopathy (NPDR).

## Methods

### Study Design

The study adopted a cross-sectional study design involving Persons living with Diabetes (PLWD).

### Study setting and participants

The study was conducted in Korle Bu Teaching Hospital (KBTH), located in Ablekuma South metropolitan district, Accra. KBTH was established in 1923 and has over 2000 bed capacity coupled with 21 clinical and diagnostic departments. Currently, the KBTH is the third biggest referral Centre in Africa with an average outpatient attendance of 1,500 with about 150 inpatient admissions daily [13]. The endocrine clinic is a subunit of the Department of Medicine and Therapeutics. Being one of the units of the leading tertiary referral Centre in Ghana, it attracts referral cases from all the regions in the country. The endocrine clinic provides medical care and consultation for patients with diabetes mellitus, hypertension, and thyroid conditions. It has an annual turnover of about 2,220 and a monthly turnover of 185 diabetic cases [13]. The clinic has two endocrinologists, one diabetologist, five physician consultants, two junior doctors, eight nurses, two health care assistants, and two orderlies.

### Sample size estimation

The study adopted the Cochran formula as cited by Lopes and colleagues for calculating sample size from a finite population. The sample was derived using the formula $n=\frac{{z}_{\alpha /2}P(1-P)}{{e}^{2}}$ where n is the desired sample size, P is the assumed 50 % prevalence rate for eye care health services utilization among diabetic patients and e is the acceptable 5 % margin error. The calculated sample size was 384. Based on Montesinos-López et al., a recommendation for finite population size (< 10,000) [14], the sample size was corrected by using the formula $\frac{n}{1+\frac{n}{N}}$ where *n* is the calculated sample 384 size and *N* is the 2220 finite population. The corrected sample size derived was 327, adjusting for a 10 % non-response rate, the final minimal sample size was 360. The study achieved a 100 % response rate.

### Data collection method

 Our interest was to assess eye care health service utilization and the association with diabetic retinopathy among PLWD. A systematic sampling method was employed for this research work. This was done on the assumption that the research was a hospital-based where healthcare service is being provided based on systematic processes (In Ghana, first come first serve). At the clinic, clinical health services are provided to patients with diabetes on weekly basis. Therefore, this study used weekly attendance, divided by the total sample size to know the sampling interval (nth interval) for sampling.

The inclusion criteria was strictly for patients with diabetes visiting the endocrine clinic at the KTBH. A comprehensive list of patients who met the eligibility criteria of the study were selected from the clinic register and placed in a box. Thus, 36 patients with diabetes were randomly sampled every week between May and July 2019. For every week, a list of sampling frame was generated from the total patients attending the eye clinic. The list was entered in Excel 2016 and the random selection command was used to select participants for the study every week. A unique identification number was used to differentiate one patient from the other. An identified respondent was selected and about 10–20 min estimation of time was spent on each respondent. All participants were provided with an information sheet about the study and their informed consent were obtained. For those who could not read or write, interpretation in local language was provided to their optimum understanding.

### Study variables

**Fig. 1:**
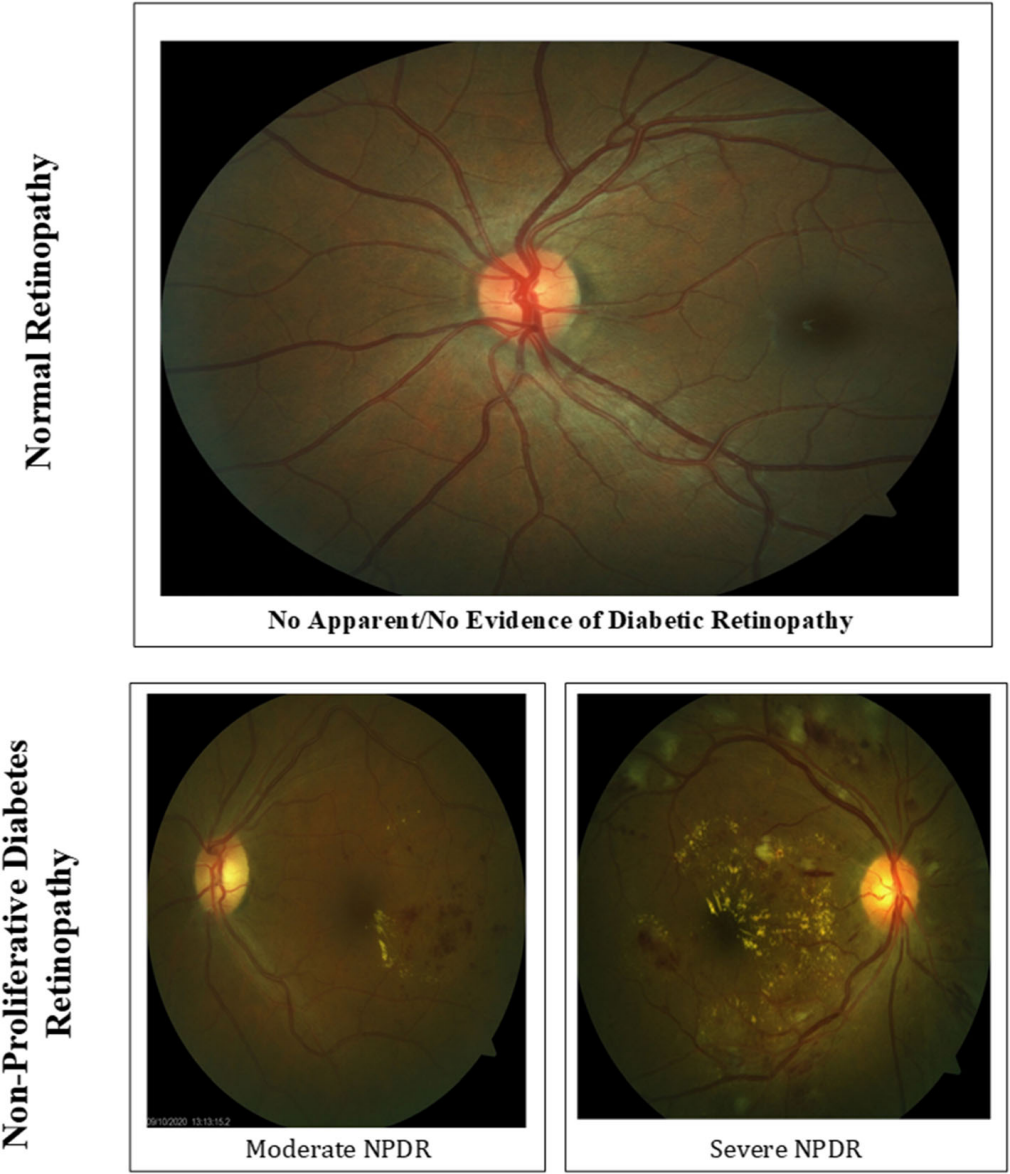
.

The secondary and primary outcome variables involved in the study were Utilization of Eye Health Service (UEHS) and Non-Proliferative Diabetic Retinopathy (NPDR) respectively. UEHS was subjectively measured by asking participants; *Have you visited an eye clinic in the last year/12 months for eye screening?* with responses 1 “Yes” 0 “No”. For NPDR, clinical measurement using the ZEISS Visucam 524/224 Fundus Camera was employed, after the client has undergone dilation of the pupil to enable the image of the retina to be captured. The classification of the retinal abnormalities was done by using the International Council of Ophthalmology Guidelines for Diabetic Eye Care with the updated version as presented in Table 1 [15]. We identified patients with no apparent DR, moderate NPDR, and severe NPDR with CSME which were coded as 0, 1, and 2 respectively as presented in Fig. 1. We further recategorized into no apparent DR (0) and NPDR (1) with Clinically Significant Macular Edema (CSME) to reflect a dummy variable.

**Table 1:** Classification of Diabetic Retinopathy and Diabetic Macular Edema

Diabetic retinopathy	Findings observable on retinal image
No apparent DR	No abnormalities
Moderate Non-proliferative DR	Microaneurysms and other signs less than severe Non-proliferative DR: • Dot and blot hemorrhages • Hard exudates • Cotton wool spots • Venous beadings
Severe Non-proliferative DR	Moderate non-proliferative DR with any of the following: • Intraretinal hemorrhages • Definite venous beading (in 2 quadrants); • Intraretinal microvascular abnormalities • No signs of proliferative retinopathy
**Diabetic macular edema (DME)**	**Findings observable on retinal image**
CSME	Retinal thickening in the macula that does not involve the central subfield zone that is 1mm in diameter or retinal thickening in the macula that does involve the central subfield zone that is 1mm in diameter

Source: International Council of Ophthalmology Guidelines for Diabetic Eye Care, the updated version

### Data analysis

Descriptive and inferential analyses were employed in this study. Bivariate descriptive statistics involving chi-square and t-test of equality proportion and means respectively were employed to assess significance association. To assess significant factors that predispose participants to our secondary outcome variable (eye service utilization), due to the dummy nature, we adopted Poisson and Probit regression analysis. These models were individually adapted to respectively assess the prevalence ratio and the log-likelihood ratio.

 The study objective was to assess the association between UEHS and the presence of diabetic retinopathy. To achieve this objective, we firstly employed pairwise and phi correlation (fourfold correlational analysis) to assess the strength of the relationship between UEHS and the presence of diabetic retinopathy (ordinal and binary respectively). Inferential analysis involving ordered logistic was performed to assess the log-likelihood of experiencing severe non proliferative diabetic retinopathy. In addition, Poisson and Probit regression analysis were applied to assess the association between the UEHS and the presence of DR. Stata 16 was used to perform the analyses and a p-value ≤ 0.05 was deemed significant.

## Results

The ratio of type 1 to type 2 diabetes among the participants was approximately 1:10 with an approximately 1:1 sex ratio. The age ranged from 18 to 87 years with an overall mean ± standard deviation of 44.6 ± 16.99 years. Eye care health service utilization (UEHS) prevalence within the past year among the participants was 21.7 %(95 %CI = 17.7–26.2). Independence test of proportion and means equality showed that the following variables; type of diabetes, sex, age, occupation, mode of payment for health services, years of diagnosis, current blood glucose, attended to by health professional during last visits and visitation to eye clinic within the past 12 months were independently associated with UEHS (*p* < 0.05; Table 2).

**Table 2:** Utilization of eye health service among participants by socio-demographic characteristics

Variable	Utilization of eye health services			χ^**2**^
	Yes	No	Total	
	P[95%CI]	P[95%CI]		
	21.7[17.7-26.2]	78.3[73.7-82.3]	***N***= 360	
	%	%	N	
**Diabetes type**				55.14***
Type 1 diabetes	74.2	25.8	31	
Type 2 diabetes	16.7	83.3	329	
**Sex**				5.23*
Male	27.2	72.8	162	
Female	17.2	82.8	198	
**Age [Mean±SD]**	44.63(20.59)	44.58(15.89)	44.60(16.99)	12.56*
≤29	30.3	69.7	76	
30-39	23.4	76.6	77	
40-49	17.7	81.3	63	
50-59	8.4	91.6	71	
60+	27.0	73.0	74	
**Educational level**				6.87
None	13.9	86.1	36	
Primary	15.3	84.7	85	
Secondary	28.8	71.2	118	
Tertiary	21.5	78.5	121	
**Occupation**				16.30***
Government Employee	23.5	76.5	85	
Self-Employed	26.3	73.7	114	
Student	40.6	59.4	32	
Retired	13.6	86.4	59	
Unemployed	10.0	90.0	70	
**Mode of payment for health services**				59.60***
Out of pocket	27.7	72.3	83	
NHIS	12.5	87.5	232	
Out of pocket and NHIS	67.6	32.4	37	
**Marital status**				7.33
Single	34.6	65.4	52	
Married	17.0	83.0	165	
Divorced	22.7	77.3	66	
Widowed	22.1	77.9	77	
**Years of diagnosis**				16.66***
< 6 months	47.6	52.4	21	
6 months - 1 year	28.3	71.7	113	
1+ years	15.9	84.1	226	
**Current blood glucose** **(mmol/L)**				6.53*
Below 5.6	40.0	60.0	30	
5.6 - 6.9	20.8	79.2	106	
7+	19.6	80.4	224	
**Time taken to health facility**				0.93
<2 hours	18.2	81.8	33	
2-4 hours	18.8	81.3	80	
5+ hours	23.1	76.9	247	
**Attended to by health professional during the last visit**				4.39*
Yes	21.1	78.9	355	
No	60.0	40.0	5	
**Visitation to Eye clinic within the past 12 months**				9.67**
Yes	33.7	66.3	86	
No	17.9	82.1	274	
**Know of any other eye clinic to visit**				2.45
Yes	24.4	75.6	217	
No	17.5	82.5	143	

NOTE: P represent the estimated prevalence rate. NHIS represent National Health Insurance Scheme.

*P*-value notation: ^*^p-value<0.05, ^**^p-value≤0.01, ^***^p-value≤0.001

The inferential analysis depicts that, type of diabetes, age, educational level, mode of payment for healthcare services, marital status, years of diagnosis, and current blood glucose significantly influenced UEHS. Interestingly, UEHS by people with type 1 diabetes was approximately 6-folds higher compared with type 2 diabetic patients [aPR(95 %CI) = 5.61(2.63–11.95)]. Meanwhile, Probit regression showed, type 1 diabetes patients increased the prevalence of UEHS by over 2-folds [aβ(95 %CI = 2.40(1.52–3.29)]. Patients aged ≤ 29 years were more likely to utilize eye care health services while higher education predisposes patients to UEHS (Table 3).

**Table 3:** Poisson and Probit regression analysis showing an association between type of diabetes and eye care health service utilization adjusting for demographic characteristics among patients visiting an eye clinic

Variable	Poisson	Probit
	aPR[95CI]	aβ[95CI]
**Diabetes type**		
Type 2 diabetes	**1.00**	**1.00**
Type 1 diabetes	5.61[2.63-11.95]***	2.40[1.52-3.29]***
**Sex**		
Male	**1.00**	**1.00**
Female	0.95[0.64-1.42]	-0.27[-0.72-0.19]
**Age**		
≤29	**1.00**	**1.00**
30-39	0.52[0.27-0.96]*	-0.62[-1.34-0.09]
40-49	0.50[0.23-1.05]	-0.74[-1.54-0.04]
50-59	0.23[0.09-0.58]**	-1.12[-2.02--0.21]*
60+	0.77[0.39-1.52]	1.82[-1.01-0.54]
**Educational level**		
None	**1.00**	**1.00**
Primary	3.30[0.72-13.72]	1.04[-0.40-2.49]
Secondary	6.16[1.86-20.40]**	2.14[0.88-3.42]***
Tertiary	4.76[1.60-14.21]**	1.82[0.66-2.98]**
**Occupation**		
Government	**1.00**	**1.00**
Self-Employed	1.14[0.61-2.14]	0.32[-0.33-0.98]
Student	0.59[0.17-2.03]	-0.38[-1.69-0.93]
Retired	1.12[0.36-3.47]	0.51[-0.55-1.57]
Unemployed	0.85[0.25-2.93]	0.08[-0.92-1.08]
**Mode of payment for health services**		
NHIS	**1.00**	**1.00**
Out of pocket	2.35[1.27-4.32]**	0.92[0.37-1.47]***
Out of pocket and NHIS	3.83[2.01-7.30]***	2.26[1.46-3.06]***
**Marital status**		
Widowed	**1.00**	**1.00**
Single	1.11[0.49-2.50]	-0.21[-1.16-0.73]
Married	1.60[0.81-3.15]	0.03[-0.61-0.67]
Divorced	2.33[1.05-5.19]*	0.39[-0.34-1.12]
**Years of diagnosis**		
<6 months	**1.00**	**1.00**
6 months - 1 year	2.35[1.27-4.37]**	1.03[0.22-1.82]**
1+ years	1.35[0.89-2.05]	0.35[-0.13-0.83]
**Current blood glucose (mmol/L )**		
Below 5.6	**1.00**	**1.00**
5.6 - 6.9	1.25[0.70-2.24]	0.44[-0.30-1.18]
7+	2.65[1.25-5.62]**	0.93[0.13-1.74]*
**Time taken to health facility**		
<2 hours	**1.00**	**1.00**
2-4 hours	1.19[0.55-2.57]	0.14[-0.56-0.83]
5+ hours	1.11[0.49-2.51]	0.15[-0.54-0.84]
**Attended to by health professional during last visits**		
Yes	**1.00**	**1.00**
No	2.61[0.77-8.93]	1.71[0.37-3.05]*
**Visitation to Eye clinic within the past 12 months**		
Yes	**1.00**	**1.00**
No	1.11[0.73-1.67]	0.38[-0.15-0.90]
**Know of any other eye clinic to visit**		
Yes	**1.00**	**1.00**
No	0.90[0.55-1.47]	-0.12[-0.61-0.37]

Note: NHIS represent National Health Insurance Scheme. aPR and aβ denote adjusted prevalence Ratio from Poisson regression and adjusted coefficient from Probit regression.

*P*-value notation: ^*^p-value<0.05, ^**^p-value≤0.01, ^***^p-value≤0.001

Having secondary and tertiary education levels significantly increased the prevalence ratio of UEHS by approximately 2 times compared with no formal education [aβ(95 %CI = 2.14(0.88–3.42) and 1.82(0.66–2.98) respectively]. In addition, out of pocket health services as a mode of payment among participants significantly increased UEHS compared with participants who only pay with NHIS [aPR(95 %CI) = 2.35(1.27–4.32)]. Surprisingly, divorced participants were over 2-folds likely to UEHS compared with their counterparts who were widowed [aPR(95 %CI) = 2.33(1.05–5.19)], however, divorce does not significantly increase the prevalence ratio [aβ(95 %CI = 0.39(-0.34-1.12)] (Table 3).

Patients who were diagnosed within 6–24 months doubles the likelihood and increased the prevalence ratio of UEHS compared with patients diagnosed less than 6 months [Poisson estimate; aPR(95 %CI) = 2.35(1.27–4.37) and Probit estimate; aβ(95 %CI) = 1.03(0.22–1.82)]. Patients with a blood glucose level of over 7mmol/L were over 2 times likely to utilize eye health service compared with 5.6 mmoL/L or below blood glucose level patients [aPR(95 %CI) = 2.65(1.25–5.62)] (Table 3).

The overall prevalence of NPDR was 65.0(95 %CI = 59.9–69.8), meanwhile, the prevalence of moderate NPDR with CSME and severe NPDR with CSME was 41.1(95 %CI = 36.1–46.3) and 23.9(95 %CI = 19.8–28.6) respectively. The overall prevalence of NPDR was significant and negatively correlated with UEHS (Table 4).

**Table 4:** Prevalence of Non-proliferative diabetic retinopathy and correlation with utilization of eye health service among patients visiting eye clinic

Variable	Prevalence	Correlation with eye care health service utilization
**Diabetic retinopathy-Ordinal**		-0.20***^a^
No apparent DR		
Moderate NPDR with CSME	41.1[36.1-46.3]	
Severe NPDR with CSME	23.9[19.8-28.6]	
**Diabetic retinopathy**		-0.15**^b^
No apparent DR		
NPDR	65.0[59.9-69.8]	

Note: DR denote Diabetes Retinopathy; NPDR denote No-Proliferative Diabetes Retinopathy; CSME denote Clinically Significant Macular Edema. Superscript a and b represent Pairwise and Phi correlation analysis.

Note: *P*-value notation: ^**^p-value≤0.01, ^***^p-value≤0.001

The association between UEHS and the presence of Non-proliferative diabetic retinopathy (NPDR) showed that the overall UEHS was significantly associated with severe NPDR with CSME, however, the overall presence of NPDR was statistically insignificant. The analysis depicts that, non-UEHS among PLWD doubles the likelihood of experiencing severe NPDR with CSME compared with their counterparts who utilize eye care health service [aOR(95 %CI) = 2.05(1.03–4.08)]. Meanwhile, the prevalence of NPDR with CSME among patients per non-EHSU was insignificantly 12 % higher compared with patients with EHSU [aPR(95 %CI) = 1.12(0.89–1.41)]. In addition, non-UEHS among patients increased the chance of experiencing NPDR with CSME by 25 %, however, statistically not significant [aβ(95 %CI = 0.25(-018-0.68)] (Table 5).

**Table 5:** Association between eye care health service utilization and the occurrence of Nonproliferative diabetic retinopathy among patients visiting eye care clinic

Variable	Model 1-Ordered	Model 2-Binary	
	Ordered Logistic	Poisson	Probit
	aOR[95%CI]	aPR[95CI]	aβ[95CI]
**Healthcare utilization**			
Yes	**1.00**	**1.00**	**1.00**
No	2.05[1.03-4.08]**	1.12[0.89-1.41]	0.25[-0.18-0.68]
**Diabetes type**			
Type 2 diabetes	**1.00**	**1.00**	**1.00**
Type 1 diabetes	0.47[0.14-1.54]	0.76[0.46-1.26]	-0.36[-1.03-0.29]
**Age**			
≤29	**1.00**	**1.00**	**1.00**
30-39	2.38[1.35-4.17]**	1.48[1.20-1.98]**	0.62[0.17-1.06]**
40-49	3.30[1.57-6.90]**	1.49[1.08-2.04]**	0.65[0.13-1.16]*
50-59	4.27[1.73-10.56]**	1.40[1.00-1.96]*	0.50[-0.02-1.04]
60+	10.16[4.58-22.52]***	1.87[1.37-2.56]***	1.25[0.65-1.86]***
**Educational level**			
None	**1.00**	**1.00**	**1.00**
Primary	0.95[0.40-2.26]	0.87[0.63-1.20]	-0.31[-1.09-0.44]
Secondary	0.96[0.44-2.10]	0.70[0.53-0.92]**	-0.69[-1.39-0.01]
Tertiary	2.01[0.97-4.14]	0.88[0.68-1.14]	-0.22[-0.94-0.49]
**Mode of payment for health services**			
Out of pocket and NHIS	**1.00**	**1.00**	**1.00**
NHIS	7.35[3.95-13.66]***	1.96[1.47-2.60]***	1.03[0.67-1.39]***
Out of pocket	2.10[1.07-4.14]*	1.58[1.11-2.25]**	0.66[0.06-1.26]*
**Years of diagnosis**			
1+ years	**1.00**	**1.00**	**1.00**
< 6 months	2.23[0.73-6.79]	1.10[0.72-1.67]	0.21[-0.54-0.96]
6 months - 1 year	2.54[1.49-4.29]***	1.17[0.95-1.44]	0.34[-0.04-0.73]
**Current blood glucose (mmol/L)**			
7+	**1.00**	**1.00**	**1.00**
Below 5.6	4.36[1.39-13.68]**	1.11[0.77-1.59]	0.40[-0.30-1.11]
5.6 - 6.9	0.83[0.49-1.41]	0.98[0.72-1.11]	-0.13[-0.55-0.28]

NOTE: NHIS represent National Health Insurance Scheme. aPR and aβ denote adjusted prevalence Ratio from Poisson regression and adjusted coefficient from Probit regression.

*P*-value notation: ^*^p-value<0.05, ^**^p-value≤0.01, ^***^p-value≤0.001

## Discussion

Persons living with diabetes (PLWD) could be blind with DR [16] and if detecting it early depends on eye health service utilization, then the prevalence of UEHS among participants in this current study is low. In this study, UEHS among PLWD was a little more than one-fifth [21.7 %(95 %CI = 17.7–26.2)]. This prevalence was 6.4 % higher compared with what Benoit and colleagues established (15.3 %) [16] and almost half of what Akuffo et al. established in South Africa (49.0 %) [17]. In addition, this proportion is 1.1 % less and statistically not different compared with what was established in Hawassa city, South Ethiopia [18]. The difference between our current study compared with other scholars could be due to the different populations involved, the type of study design, and the jurisdiction of the participants involved.

Factors associated with UEHS as identified in this current study are in line with previous studies [17–21]. In this current study, the type of diabetes was a key indicator and was significantly associated with UEHS. We found that adjusting for other significant factors, the likelihood of UEHS among type 1 diabetes patients was higher compared with those who had type 2 diabetes mellitus. Type 1 diabetes is in relatively younger persons, who are insulin dependent and need to visit the health facility for management of their insulin treatment. Type 2 diabetes is most often found in relatively older adults, who may be non-insulin dependent and can go for a long time without obvious ill-effect of their illness condition and may be unaware of the implication of the condition that may affect their eyes and may not understand the importance of retinopathy screening [21, 22].

### Association between utilization of eye health service and Non-proliferative diabetic retinopathy

Globally prevalence of diabetes is on the rise both in high-income countries and in Low-Middle-Income-Countries (LMICs) and its associated implication are a major public health issue requiring effective eye health screening to prevent the associated vascular complication like retinopathy [23].

This current study revealed that more than two-thirds of patients attending to the endocrine clinic of KBTH had DR (65 %) comprising 41 % moderate NPDR with CSME and 32.9 % severe NPDR with CSME. Globally, 34.6 %, 6.96 %, and 6.81 % overall prevalence rates of DR, PDR, and DME respectively exist [24] which are less than the findings in this study. In addition, these current prevalence rates were significantly higher than what Song and colleagues established in their systematic review in China. They found a pooled prevalence of 18.4 %, 15.0 %, and 1 % respectively for DR, NPDR, and PDR [25]. Elsewhere in the United States, the estimated crude prevalence rates for retinopathy was 40.3 % [26] which is 24.7 % less compared with our current study. Meanwhile, a similar hospital study that adopted the design nature of our study established 13 % DR among diabetic patients in Ethiopia [27]. The high variation between our current prevalence rate and that of Chisha and colleagues could be the health systems structure. Our study was conducted in a National referral health facility in Ghana (KBTH) where patients are referred from all corners of the country with a total bed capacity of 2000 whiles Chisha et al. conducted their study in a zonal health facility (Arba Minch General Hospital) with only 200 bed capacity and 251 health professionals [28].

A significant negative correlation exists between DR and UEHS, meaning that participants with DR were identified as those who do not uptake UEHS. This observation is in contradiction to a finding which established that diabetes increased health service utilization [29]. However, health service utilization may be relatively high, but specific uptake of UEHS  may not be high as demonstrated in our analysis. The reasons accounting for this poor UEHS in our study abound. Lack of knowledge on the asymptomatic nature of DR and its effects on vision influence patient’s unwillingness to undergo eye care screening services, and this could be a probable explanation. In addition, there are no ophthalmoscopes at the various consulting rooms to enable physicians to carry out annual eye screening services for patients.

Overall, we have determined that poor UEHS among PLWD doubles the likelihood of experiencing severe NPDR with CSME. This finding affirms the assertion that comprehensive dilated fundal examination of the eye at least once a year is key in detecting DR which may not have any symptoms at first but finding it early can help save patients from irreversible blindness [30].

## Limitation

Recall bias may influence the prevalence of UEHS since patients were asked to recall over the past 12 months whether they visited an eye clinic for screening. This study adopted a cross-sectional study and that it doesn’t show a causal relationship between UEHS and the occurrence of DR among the patients.

## Conclusions

DR plays an integral role in causing blindness among patients as established by WHO [31]. This study identified a high rate of PLWD not utilizing the eye health service at least once a year. Approximately, one-fifth of the participants utilized eye health service over the past year and was highly influenced by type of diabetes and increasing age. Patients in their middle age with Type 2 diabetes were found less likely to utilize eye health services compared with their younger counterparts with Type 1 diabetes. There was a negative relationship between DR and UEHS and that non-UEHS among patients doubles the likelihood of experiencing severe NPDR with CSME. Nearly all PLWD will have some form of retinopathy in their lifetimes and routine eye screening and good diabetes control can protect them from DR and its harmful consequences [31]. Early detection and management of DR among PLWD are essential in reducing impairment and disability. Thus, structured health education and eye screening intervention are key to improving UEHS.

## Data Availability

The datasets used and/or analyzed during the current study are available from the corresponding author on reasonable request.

## Abbreviations

aOR: Adjusted Odd Ratio
aPR: Adjusted Prevalence Ratio
CI: Confidence Interval
DR: Diabetes retinopathy
NPDR: Non-Proliferative Diabetes Retinopathy
CSME: Clinically Significant Macular Edema
WHO: World Health Organization
